# Defective Localization With Impaired Tumor Cytotoxicity Contributes to the Immune Escape of NK Cells in Pancreatic Cancer Patients

**DOI:** 10.3389/fimmu.2019.00496

**Published:** 2019-04-09

**Authors:** Seon Ah Lim, Jungwon Kim, Seunghyun Jeon, Min Hwa Shin, Joonha Kwon, Tae-Jin Kim, Kyungtaek Im, Youngmin Han, Wooil Kwon, Sun-Whe Kim, Cassian Yee, Seong-Jin Kim, Jin-Young Jang, Kyung-Mi Lee

**Affiliations:** ^1^Department of Biochemistry and Molecular Biology, Korea University College of Medicine, Seoul, South Korea; ^2^Department of Surgery and Cancer Research Institute, Seoul National University Hospital, Seoul National University College of Medicine, Seoul, South Korea; ^3^Department of Melanoma Medical Oncology and Immunology, MD Anderson Cancer Center, Houston, TX, United States; ^4^Precision Medicine Research Center, Advanced Institutes of Convergence Technology, Seoul National University, Suwon, South Korea; ^5^Center for Bio- Integrated Electronics, Simpson Querrey Institute, Evanston, IL, United States

**Keywords:** pancreatic cancer, cancer immunobiology, cellular immunology, immunotherapy, chemokines

## Abstract

Tumor-infiltrating lymphocytes (TILs), found in patients with advanced pancreatic ductal adenocarcinoma (PDAC), are shown to correlate with overall survival (OS) rate. Although majority of TILs consist of CD8^+^/CD4^+^ T cells, the presence of NK cells and their role in the pathogenesis of PDAC remains elusive. We performed comprehensive analyses of TIL, PBMC, and autologous tumor cells from 80 enrolled resectable PDAC patients to comprehend the NK cell defects within PDAC. Extremely low frequencies of NK cells (<0.5%) were found within PDAC tumors, which was attributable not to the low expression of tumor chemokines, but to the lack of chemokine receptor, CXCR2. Forced expression of CXCR2 in patients' NK cells rendered them capable of trafficking into PDAC. Furthermore, NK cells exhibited impaired cell-mediated killing of autologous PDAC cells, primarily due to insufficient ligation of NKG2D and DNAM-1, and failed to proliferate within the hypoxic tumor microenvironment. Importantly, these defects could be overcome by *ex-vivo* stimulation of NK cells from such patients. Importantly, when the proliferative capacity of NK cells *in vitro* was used to stratify patients on the basis of cell expansion, patients whose NK cells proliferated <250-fold experienced significantly lower DFS and OS than those with ≥250-fold. *Ex-vivo* activation of NK cells restored tumor trafficking and reactivity, hence provided a therapeutic modality while their fold expansion could be a potentially significant prognostic indicator of OS and DFS in such patients.

## Introduction

Pancreatic ductal adenocarcinoma (PDAC) is one of the most lethal human malignancies (1, 2). Majority of patients with PDAC are diagnosed with distant metastasis or locally advanced stages, rendering them surgically inoperable at the time of diagnosis (3). Although patients with metastases from colorectal or breast cancer have reported improved outcomes with the advent of new chemotherapeutic agents over the last decade, there has not been much increase in survival of patients with pancreatic cancer. Anatomical location, delayed diagnosis, and chemo-resistance, associated with a stromal compartment that serves as a barrier for chemotherapeutic drugs, are key factors contributing to the poor prognosis observed in patients with PDAC (4). Furthermore, PDAC has been shown to be inherently immunosuppressive, producing TGF-β, IL-10, IDO, and MMPs (5–9), thereby allowing their escape from immune surveillance. For example, the PDAC tumor microenvironment favors the expression of co-inhibitory ligands, PDL-1 and PDL-2, and inhibits HLA class I expression in tumor cells (10–12) while promoting the emergence of regulatory T cells (Treg) and tumor-associated macrophages (TAM) that secrete immunosuppressive cytokines (5, 13). Moreover, pancreatic carcinoma are supplied with insufficient and aberrant blood vessels, and are hence extremely hypoxic. Under low oxygen conditions, growth of stellate cells, the major fibroblastic cells of the pancreas co-residing with tumor cells, is significantly accelerated (14).

Despite the severe desmoplastic and immunosuppressive nature of PDAC microenvironment, a significant level of Tumor-infiltrating lymphocytes (TIL) infiltration has been found in patients with PDAC that correlates with clinical prognosis (15, 16). A high level of CD8^+^ and CD4^+^ T cells, together with a low number of Tregs, was found to be associated with improved survival in such patients (17). However, most of the activated T cells, localized to PDAC, express an array of co-inhibitory receptors, including PD-1, LAG-3, TIM3, and CTLA-4, which can induce tolerance and exhaustion of Antigen-specific T cells after the initial recognition of tumors (9, 18–21).

NK cells are bone marrow-derived large granular lymphocytes shown to provide the body's first line of defense (22, 23). NK cell lytic functions are regulated by an array of activating and inhibitory receptors on the cell surface, leading to the release of cytotoxic granules containing perforin and granzymes (24). Activating receptors in charge of tumor cytolysis in NK cells include NKG2D (Natural killer group2, member D), DNAM-1(DNAX accessory molecule-1), and NCR (Natural Cytotoxicity Receptors, NKp30, NKp44, NKp46) while KIR (Killer inhibitory receptors) and CD94-NKG2 heterodimers serve as inhibitory receptors (25, 26). It has been shown previously that progression of pancreatic cancer is closely associated with dysfunctional circulating NK cells in PBMC (27). However, the presence of NK cells within pancreatic tumors and their role in the progression of PDAC remains unclear. To address these issues, we obtained tumor and peripheral blood samples from such patients undergoing surgical resection, and performed a detailed analysis of NK cell frequency and their proliferation profile. Furthermore, we isolated tumor cells from the patients to assess anti-tumor function of the NK cells in autologous settings, and attempted to correlate these parameters with clinically annotated findings.

## Materials and Methods

### Cell Lines and Cell Culture

K562 (human erythroblastoid cell line; ATCC, Manassas, VA) and Jurkat, indicated as KL-1 (human T lymphoblast line; Korean Cell Line Bank, Seoul, Korea) were cultured in RPMI 1640 medium supplemented with 10% FBS, 100 U/ml penicillin, and 100 U/ml streptomycin. PANC-1, MIA PaCa-2 (human pancreatic cancer cell line; ATCC), and Hep3B (human hepatocellular carcinoma cell line; ATCC) were maintained in Dulbecco's modified Eagle's medium (DMEM) supplemented with 10% FBS, 100 U/ml penicillin, and 100 U/ml streptomycin. Human PBMCs were isolated from healthy donors and patients with pancreatic cancer. The Epstein-Barr virus (EBV)-transformed lymphoblastoid B cell line (LCL) was derived from PBMCs with EBV supernatant of B95-8 cells. For hypoxic treatments, cells were cultured in an anaerobic incubator (VISION CO2 O2 INCUBATOR, Vision Scientific Co., Ltd.). All studies containing human subjects were approved by the INSTITUTIONAL REVIEW BOARD OF KOREA UNIVERSITY with donor's consent (1040548-KU-IRB-16-103-A-2).

### Patient Characteristics

A total of 80 patients were enrolled from May 1, 2015 to July 31, 2016. Patient profiles describing age, sex, percentage of neoadjuvant therapy, primary tumor location, and TNM stage are listed in Table 1.

**Table 1:** Patients' characteristics and TNM staging of pancreatic cancers.

**Characteristics**	**Cases**	**%**
**GENDER**		
Male	49	61.3
Female	31	38.7
**AGE**		
≤65	30	37.5
>65	50	62.5
**PRIMARY TUMOR LOCATION**		
Head of pancreas	53	66.3
Body of pancreas	9	11.2
Tail of pancreas	14	17.5
IPMN	4	5
**TNM STAGE**		
I (A*/*B)	3	3.75
II (A*/*B)	5	6.25
Ill	71	88.75
V	1	1.25
**Total**	80	

### Preparation of Primary PDAC Tumor Cells and Isolation of TILs From Patients Undergoing Resection Surgery

Tumor specimens from 80 patients with PDAC were obtained from the Department of Surgery, SEOUL NATIONAL UNIVERSITY HOSPITAL (SNUH) and processed according to the guidelines provided by the ethics committee of SNUH (1503-093-657). Pathological features of all tissues were assessed according to WHO classification and AJCC staging (7th edition). The surgically dissected pancreatic tumor masses from patients with PDAC were washed with Phosphate-buffered saline (PBS) and minced into 3–5 mm^2^ slices, and collected in RPMI 1640 supplemented with 10% FBS, 100 U/ml penicillin, and 100 U/ml streptomycin. Sliced tumor fragments were dissociated with 750 U/ml of type IV collagenase (Worthington Biochemical Corporation, Lakewood, NJ), and incubated for 1 h at 37°C in 5% CO_2_, with rotation. After washing twice with RPMI 1640, a part of the cells was used for CD45 positive selection using magnetic-activated cell sorting, and the rest was used for culturing primary pancreatic cancer cells.

### Antibodies and Flow Cytometry

Anti-human CD2 FITC (RPA-2.10), CD8 APC (OKT8), CCR7 PerCP-Cy5.5 (3D12), CD57 PE (TB01), 2B4 PE (DM244), CD25 PE (BC96), Foxp3 PerCP-Cy5.5 (PCH101), LAG-3 PerCP-Cy5.5 (3DS223H), CD160 PE (BY55), CXCR4 APC (12G5), ICAM-1 PE (HA58), CD11a FITC (HI111), NKG2D APC (5C6), and CD69 FITC (FN50) mAbs were purchased from eBioscience. Anti-human CXCR3 PE (1C6), CCR10 PerCP-Cy5.5 (1B5), DNAM-1 FITC (DX11), CD94 FITC (HP-3D9), CD158a PE (HP-3E4), KIR-NKAT2 FITC (DX27), CD20 PerCP (L27), NKp46 APC (9E2), CD158b PE (CH-L), CD132 PE (AG184), NKp30 PE (P30-15), NKB1 FITC (CX9), and CD122 PE (Mik-b3) mAbs were purchased from BD Pharmingen. Anti-human CD3 FITC (OKT3), KLRG1 FITC (2F1), CD56 PE (HCD56), TIM3 PE (F38-2E2), CCR5 FITC (HEK/1/85a), CCR8 PE (L263G8), CD16 FITC (3G8), CXCR6 APC (K04125), CXCR2 PerCP-Cy5.5 (5E8), and CD45 Alexa700 (HI30) mAbs were purchased from BioLegend. Anti-human NKG2A PerCP (131411), NKG2C APC (134591) mAbs were purchased from R&D Systems. Purified anti-human DNAM-1 (102511), NKp30 (210845), NKp44 (253415), NKp46 (195314), and NKG2D (149810) from R&D Systems and ICAM-1 (Clone R6-5-D6) from Bio X Cell were used to inhibit the binding of receptors to their ligands. For surface staining, cells were stained with the indicated FITC-, APC-, PE-, or PerCP-conjugated mAbs in 0.1 ml FACS buffer (BD Biosciences). Flow cytometry was performed with FACSCanto II (BD Biosciences, San Diego, CA) and data were analyzed with FlowJo (Tree Star, Ashland, OR) software.

### Cytotoxicity Assay

^51^Cr-release assay was conducted according to the published protocol (28). Briefly, target cells were labeled with ^51^Cr (Perkin Elmer, Boston, MA) at 50 μCi/5 × 10^5^ cells and incubated for 4 h at 5 × 10^3^ cells/well with serial dilution of PBMCs. In some experiments, the assay was performed in presence of 5 μg/ml of blocking anti-NKG2D, anti-DNAM-1, anti-NKp30, anti-NKp44, anti-NKp46, or anti-ICAM1 mAbs or isotype controls. The γ-scintillation of supernatant was quantified using a γ-counter (Perkin Elmer). Percentage of specific lysis was measured with the following formula: 100 × (experimental release-spontaneous release)/(maximum release-spontaneous release). NK cell cytotoxicity, in hypoxic condition, was determined using the Calcein-AM release assay. Target cells were labeled with 2–4 μg/ml (titrated for each tumor cell line) of Calcein-AM (Sigma-Aldrich) for 30 min at 37°C with occasional shaking. Cells were co-cultured at the indicated effector-to-target (E:T) ratios and incubated at 37°C for 4 h. After incubation, 80 μl of the supernatant was harvested and transferred to a new plate. Samples were measured using a Hidex Sense microplate reader (Ex: 485 nm/Em: 530 nm). Percent lysis was calculated with the same formula used for the ^51^Cr release assay.

### Quantitative Real-Time Polymerase Chain Reaction (qRT-PCR) Analysis

Total RNA was extracted from the cells and tissue specimens using TRIzol reagent (Life Technologies, Carlsbad, CA) according to the manufacturer's instructions. All quantitative real-time PCR amplifications were performed using StepOnePlus (Applied Biosystems, Foster City, CA) and SYBR green supermix (Bio-Rad). Gene expression was normalized using 18S rRNA. Primer sequences used are listed in Table S1.

### Lentiviral Transduction of CXCR2 in NK Cells

Full-length human CXCR2 was obtained from Addgene (cat #66260, MA) and amplified by polymerase chain reaction. pCDH-521A lentiviral plasmids carrying CXCR2 were co-transfected with packaging plasmids into 293 FT cells using CaCl_2_, and supernatant was collected after 72 h of culture. A total of 5 × 10^6^ NK cells were seeded with virus in each well of 48-well plates with complete medium containing 8 μg/ml Polybrene (hexadimethrine bromide, Sigma-Aldrich). The following day, viral supernatant was removed and replaced with growth medium. Four days later, transduced NK cells were collected for further analysis.

### Migration Assay

Vector or CXCR2-transduced NK cells (1.5 × 10^5^ cells) were placed in 0.2 ml of complete medium in the upper chamber (5.0-μm pore) of a 24-well Transwell plate (Costar). Medium (1 mL) containing MIA PaCa-2 or primary tumor cells were placed in the lower chamber, and the plates were incubated for 4 h at 37°C. The number of cells in the lower chamber was manually counted. The percentage of CD3-FITC negative and CD56-APC positive NK cells in each well was quantified by FACSCanto II (BD Biosciences), and analyzed with FlowJo software (Tree Star).

### Expansion of NK Cells

Expansion of NK cells was performed as described previously (29). Briefly, PBMCs, prepared from peripheral blood using Ficoll-Paque PLUS (GE Healthcare, Uppsala, Sweden), were co-cultured with gamma-irradiated (100 Gy) KL-1 and LCL feeder cell lines in RPMI 1640 medium supplemented with 10% FBS and recombinant human IL-2 (500 U/ml; rhIL-2, Proleukin; Novartis, Basel, Switzerland). Medium was changed every 3 days up to 6 days, and every 4 days up to 18 days, thereafter. Fresh IL-2 was added when medium was changed during the culture. On day 6, expanded NK cells were transferred to T25 or T75 flasks at a concentration of 0.25 × 10^6^ cells/ml. The absolute number of NK cells was calculated by multiplying the total number of viable cells by the percentage of CD56^+^CD3^−^ cells, measured by flow cytometry. Fold change was determined by dividing the number of viable NK cells present at the designated day of culture by the number of viable NK cells at the beginning of culture.

### *In vivo* Tumor Challenges

Six to 9-week-old female NOD *scid* gamma (NSG) mice were purchased from Jackson laboratories, and maintained at Korea University (Seoul, Korea) animal facilities under specific pathogen-free conditions. All animal experiments were performed in accordance with national and institutional guidelines (KOREA-2017-0066-C1). Approximately, 1 × 10^7^ MIA PaCa-2 cells were subcutaneously injected into the right flank of NSG mice, followed by intravenous injection of 1 × 10^7^ expanded NK cells, 10 days later, at days 7, 14, 21, 28, 35, 42, and 49. Tumor volumes were measured for up to 50 days following immunization.

### Statistics

Statistical analysis was performed using SPSS version 23.0 (IBM, Armonk, NY). Nominal and continuous variables were compared using the χ2 tests and Student's *t* test, respectively. Survival rates were calculated using the Kaplan-Meier method, and the log-rank test was used to analyze the differences. The survival time and disease-free time were calculated from the start of surgery. Variables that were statistically significant in univariate analysis were included in multivariate analysis using the Cox proportional hazards regression. Two-sided *p* values of <0.05 were considered significant. A two-tailed Student's *t*-test was used for statistical comparison of two groups, where indicated, and *p*-values (^*^p ≤ 0.05; ^**^p < 0.01; ^***^p < 0.001) were taken as statistically significant.

## Results

### NK Cells Are Present at a Very Low Frequency in Tumors Resected From Patients With PDAC

**Figure 1:**
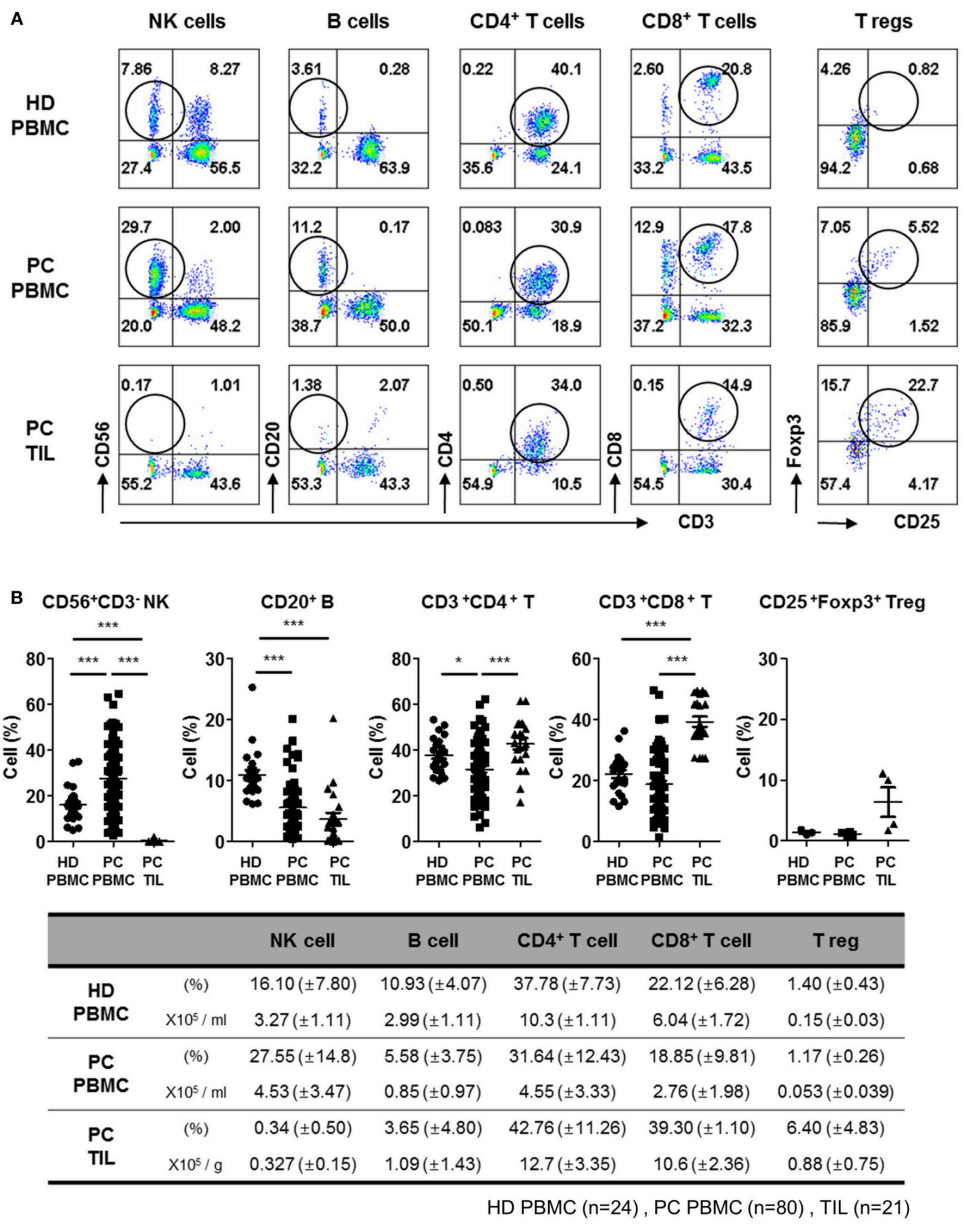
Flow cytometry analysis of tumor-infiltrating lymphocytes from patients with PDAC show lack of NK cell infiltration. **(A)** Representative flow cytometry data of lymphocyte frequency in PBMC of healthy donors, and PBMC and TIL of patients with PDAC are shown. Percentages of lymphocytes including NK, B, CD4^+^ T, CD8^+^ T, and Treg cells in TILs and PBMCs are written in each quadrangle of the graph. Treg cells were analyzed on CD3^+^CD4^+^ subset by intracellular Foxp3 staining. **(B)** The percentages of lymphocytes from individual patients are summarized as dot plots (Top) and a table showing mean ± SD (HD PBMC, *n* = 24; PDAC PBMC, *n* = 80; PDAC TIL, *n* = 21). The peripheral NK cell percentages of patients with respect to their NK cells infiltrated within TIL was marked in Red, in the Figure S4. Statistical differences between each group were calculated by Student's *t-*test (^*^p ≤ 0.05; ^***^p < 0.001).

To examine the distribution of NK cells in patients with PDAC, we first analyzed the proportion of immune cells in PBMCs isolated from newly diagnosed patients undergoing resection surgery, and compared with that of healthy donors. Patient profiles describing gender, age, percentage of neoadjuvant therapy, primary tumor location, and TNM stage are listed in Table 1. Representative flow cytometry data with gating strategies (Figures S1, S2) and individual dot graphs (Figure 1A) indicate that patients with PDAC show broad ranges of CD56^+^CD3^−^ NK cells (27.55 ± 14.8%) in PBMCs compared to healthy donors (HD); however, very little NK cells (0.34 ± 0.50%) were found within TILs of such patients. This was not likely due to the loss of surface NK markers, CD56, CD16, NKp46 during collagenase treatment of PDAC tumor specimen as NK cells isolated by Gentle MACs system also showed similar low frequency within tumor (Figure S3). The broad and relatively high percentages of NK cells in the patients' blood was likely associated with selective reduction of other lymphocytes, B, CD4, and CD8 T cells, leaving high frequency of NK cells in the blood. Indeed, our data demonstrate that the number of NK cells in the PDAC patients were not found to be significantly smaller than that of HD controls while over 50% of reduction of B, CD4 T, and CD8 T cells were reduced in the patients (Figure 1B, bottom).

In contrast, significantly high percentages of CD4^+^CD3^+^ (42.76 ± 11.26%) and CD8^+^CD3^+^ (39.30 ± 1.10%) T cells were found in TILs, as compared to those in PBMCs CD4^+^CD3^+^ (31.64 ± 12.43%) and CD8^+^CD3^+^ (18.85 ± 9.81%), indicating active localization of T cells to pancreatic tumors (17). The percentage of CD20^+^ B cells in TILs (3.65 ± 4.80%) was similar to that in PBMCs (5.58 ± 3.75%), but the percentage of CD4 Treg was 5–6 fold elevated in TILs, implying signs of immunosuppressive microenvironment. These data demonstrate that NK cells exhibit severely impaired tumor localization, distinct from other types of lymphocytes shown in patients with resectable pancreatic tumor.

### Surface Expression of CXCR2 Chemokine Receptor Is Reduced in Circulating NK Cells of Patients With PDAC

**Figure 2:**
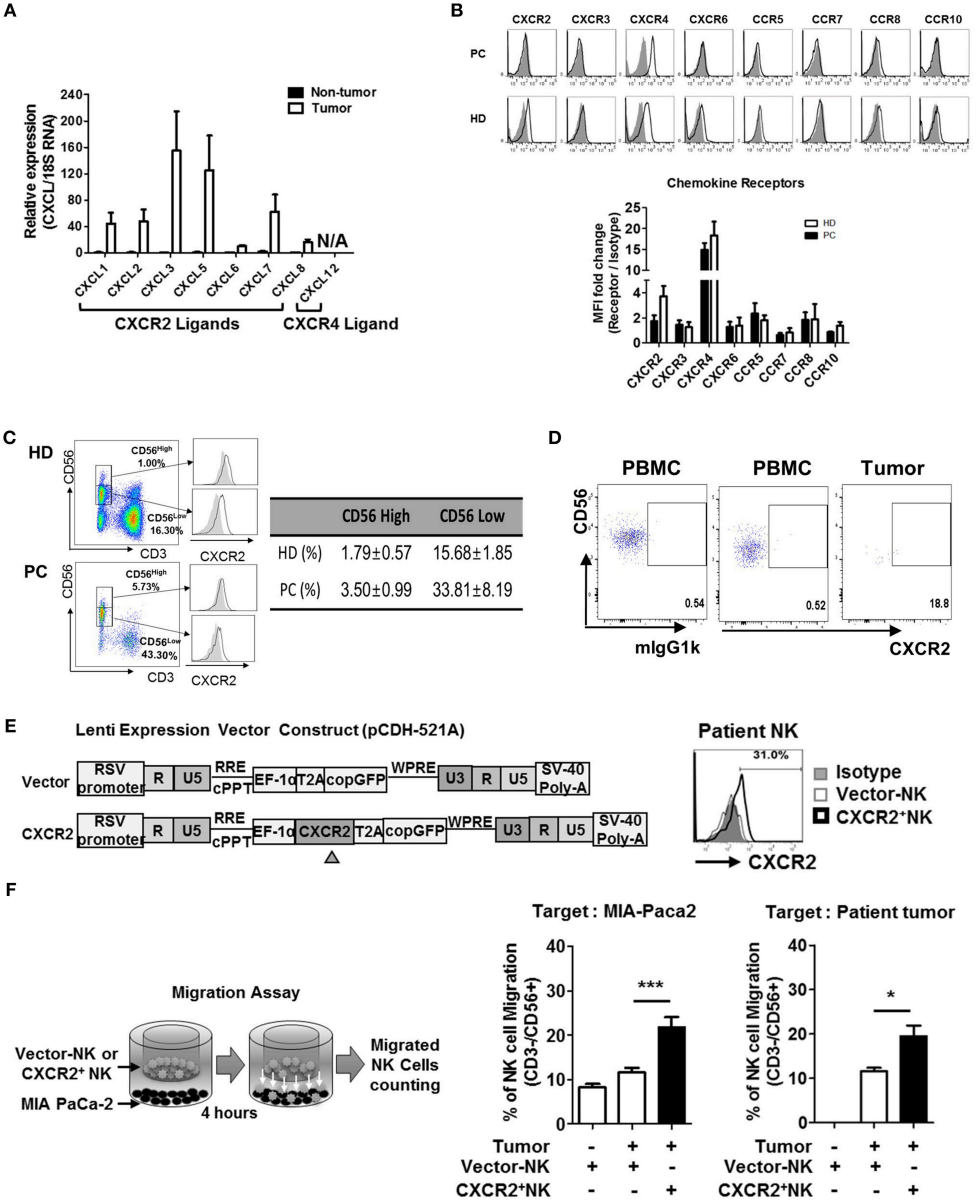
Chemokines and their receptor expression in PDAC patients. **(A)** qRT-PCR analysis of multiple chemokine expression in tumor tissues and non-tumor tissues harvested from patients undergoing resection surgery. **(B)** Flow cytometry analysis of the expression of chemokine receptors in circulating NK cells from patients vs. that from healthy donors. **(C)** CXCR2 expression on NK cells in healthy donor was gated on CD56hi and CD56lo populations (left), and their percentages in HD and PDAC patients are summarized as a table (right), with the average level of CXCR2 expression on CD56hi and CD56lo NK cells shown as bar graphs (Figure S4) **(D)** Flow cytometry analysis of CXCR2 expression on NK cells from PBMC and TIL of patients with PDAC. **(E)** Schematic diagrams of lentivirus construction expressing CXCR2 gene; CXCR2 gene was cloned upstream of RSV-copGFP cassette in the transfer vector pCDH-521A. The level of CXCR2 expression on transduced NK cells was shown by FACs. **(F)** Migration assay was performed using vector-or CXCR2-transduced patients' NK cells targeting MIA PaCa-2 (left) or Patients' tumor cells (right). Bar graph shows the percentage of NK cells migrated toward tumor targets. Data shown here are representative of three independent experiments. Statistical significance was determined by one-way ANOVA (^*^p ≤ 0.05; ^***^p < 0.001).

Based on the speculation that the low frequency of NK cells in TILs of patients with PDAC might be due to impaired chemokine secretion associated with NK cell trafficking, we analyzed the expression of chemokine ligands, produced by the pancreatic tumors, by quantitative real-time qPCR (Figure 2A, Table S1). Non-tumor tissues harvested from the same patients were used as controls. While non-tumor tissues did not show any significant level of CXCL chemokines, tumor tissues expressed high levels of CXCL3 and CXCL5 and relatively low levels of CXCL1, CXCL2, and CXCL7, similar to those reported previously (30–34). Expression of CXCL6 and CXCL8 was minimal, compared to other CXCL ligands. Furthermore, PDAC tumors did not express CXCL12, a ligand for CXCR4. These data demonstrate that lymphocytes expressing CXCR2 can efficiently localize toward pancreatic tumors. Contrary to those from HD, NK cells from patients with PDAC showed reduced surface expression of CXCR2 (Figure 2B). Downregulation of surface CXCR2 on NK cells was apparent on CD56low NK cell populations (Figure 2C, Figure S5), but was not associated with the reduced CXCR2 mRNA expression (Figure S5) or reduced the number of CD56low populations in the patients (Figure 2C). These data suggest that downregulation of CXCR2 on the cell surface of NK cells in PDAC patients is likely to occur at the post-transcription level or post-translational involving protein internalization or degradation pathways associated with immunosuppression (35–37).

It is noteworthy that the surface expression of CXCR4 on NK cells was comparable between PDAC and HD (Figure 2B), and its ligand CXCL12 was not expressed in PDAC tumors. From these data, we concluded that CXCR2, rather than CXCR4, on NK cells, might play an important role in trafficking into PDAC. In support of this notion, we observed that the small number of NK cells within PDAC TIL showed detectable CXCR2 expression (Figure 2D). To further confirm this hypothesis, we transduced resting NK cells from PDAC patients with lentivirus expressing CXCR2 and assessed their chemotaxis toward PDAC tumors. On day 3, following lentiviral transduction (Figure 2E), ~30% of patient's NK cells expressed low but detectable level of surface CXCR2. Migration assay using these NK cells demonstrated that chemotaxis toward a PDAC cell line, MIA PaCa-2, was greatly enhanced, up to 200% of the control (Figure 2F). Taken together, our data demonstrate that patients' NK cells exhibit reduced surface expression of CXCR2, responsible for tumor trafficking. Forced expression of CXCR2, by lentiviral transduction, could facilitate NK cell migration toward tumor microenvironment possessing its cognate ligands.

### Resting NK Cells Show Impaired Cytotoxic Effector Function Both in Normoxic and Hypoxic Environment

**Figure 3:**
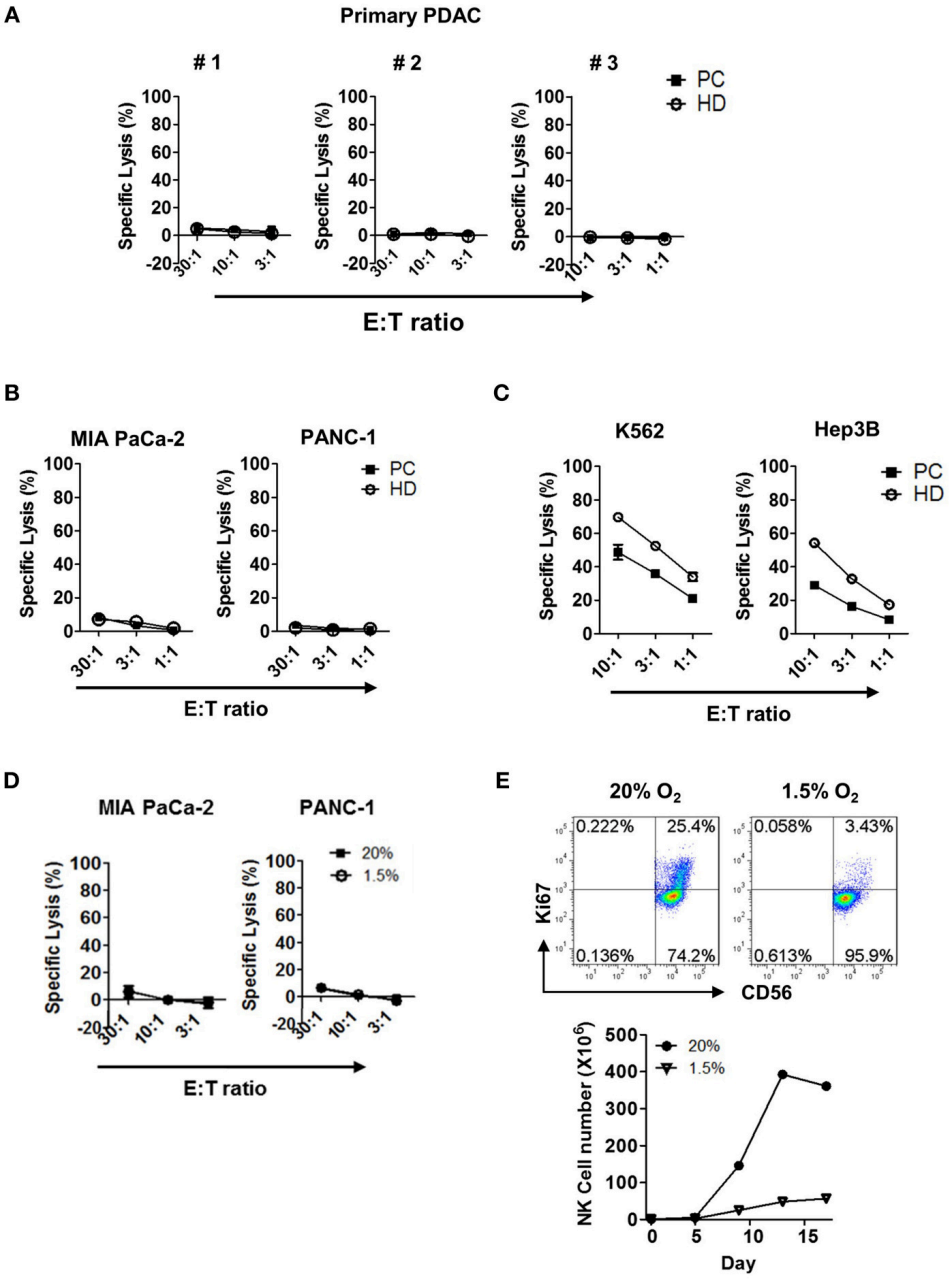
Cytotoxicity and proliferation characteristics of resting NK cells against PDAC tumor cells in both normoxic and hypoxic conditions. Cytotoxicity results of resting NK cells from patients (■) and healthy donors (°) against **(A)** primary PDAC cells, **(B)** MIA PaCa-2 and PANC-1, and **(C)** K562 and Hep3B cells are shown. Results were obtained from three different donors at indicated E:T ratios. **(D)** The cytotoxicity assays of resting NK cells from patients with PDAC against MIA Paca-2 and PANC-1 were performed under 20 and 1.5% O_2_ conditions. **(E)** NK cells isolated from healthy donors were cultured with IL-2 for 96 h, under normoxic or hypoxic conditions, were stained with Ki-67 and analyzed by flow cytometry. Data shown here are representative of five independent experiments.

Next, we examined the tumor cytotoxicity of NK cells against primary tumor cells using *in vitro* ^51^Cr-release assay. Resting NK cells present in PBMC (Figure 3A) or highly enriched up to >95% (Figure S6) from patients or HD, were incubated for 4 h with primary pancreatic tumor cells and the percentage of target lysis was determined. NK cells, from neither patients nor HD, were able to kill primary tumor cells; they were not able to kill other commercially available pancreatic cell lines, namely MIA PaCa-2 or PANC-1 (Figure 3B). This was, however, not due to the impaired anti-tumor function of NK cells, since resting NK cells from patients with PDAC showed efficient killing in hepatocellular carcinoma cell line Hep3B and myelogenous leukemia cell line K562 with ~30 and 50% efficiency, respectively, at the effector:target (E:T) ratio of 10:1 (Figure 3C). Impaired cytotoxic function of NK cells against PDAC was also shown in hypoxic tumor microenvironment of <1.5% of pO_2_ (Figure 3D). Furthermore, NK cells that managed to reach the hypoxic microenvironment of the tumor could not proliferate (Figure 3E). Collectively, these data demonstrate that resting NK cells not only fail to recognize pancreatic tumor cells for efficient killing, but also fail to proliferate in hypoxic tumor microenvironment.

### NK Cell Cytotoxicity Against Autologous and Allogeneic PDAC Tumors Is Augmented by *ex vivo* Stimulation and Expansion

**Figure 4:**
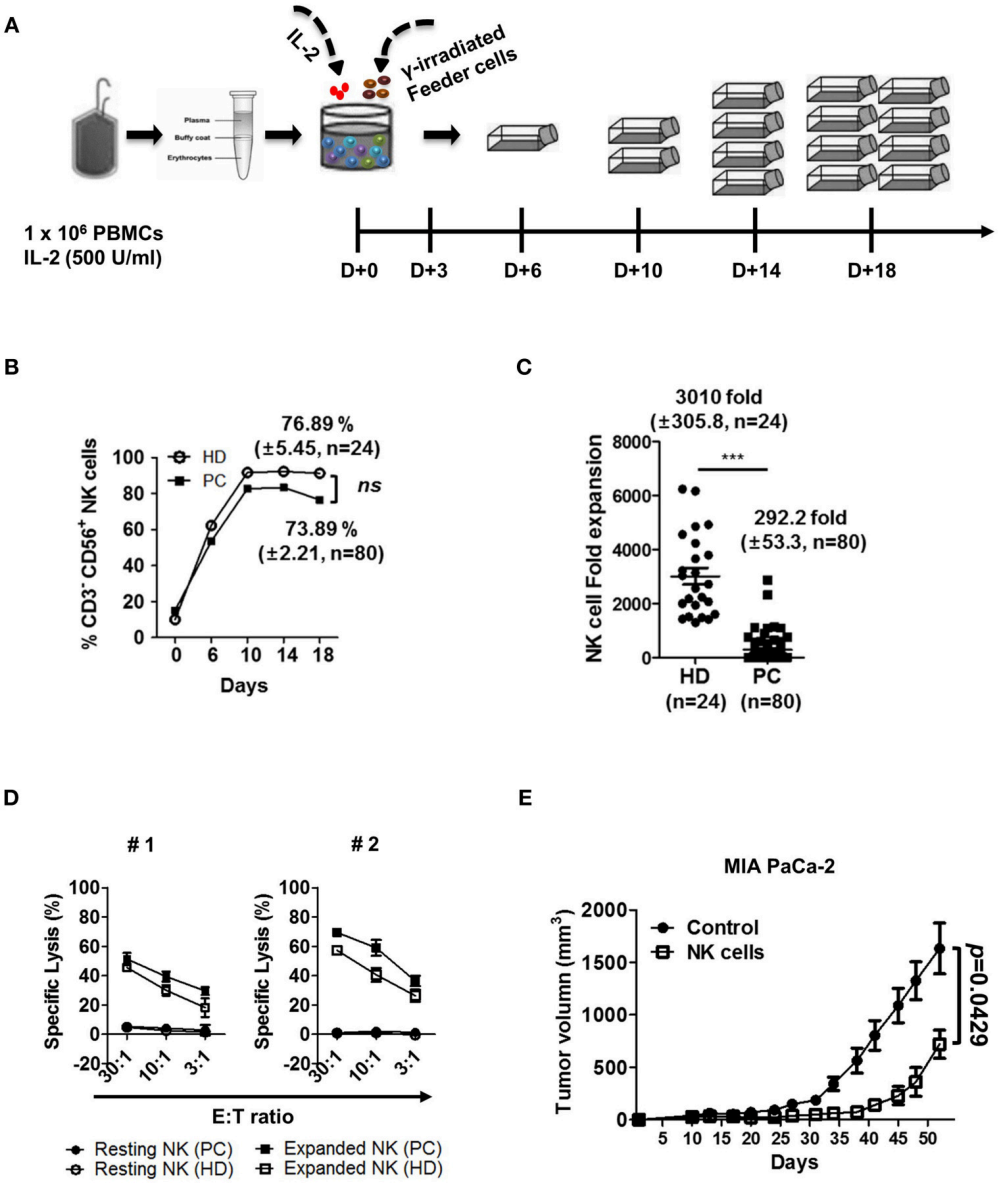
*Ex vivo* activated NK cells demonstrate strong anti-tumor cytotoxicity against autologous and allogeneic PDAC target cells. **(A)** Schematic diagram showing *ex vivo* expansion of NK cells. PBMCs were isolated from blood and cultured at a ratio of 1:0.5:0.5 with irradiated KL-1 and LCL feeder cell lines in the presence of 500 U/ml IL-2. **(B)** NK cell percentages in healthy donor and patients with PDAC are shown with respect to time. **(C)** Average fold changes of NK cells at day 18 for healthy donors and patients with PDAC are shown. **(D)** Standard 4-h ^51^Cr release assay was performed using autologous (PC) or allogeneic NK cells (HD) against autologous PDAC tumor targets. Target cells (#1, #2) used in these assays are patient-derived pancreatic cancer cell lines. Resting NK-PC (•), Resting NK-HD (°), Expanded NK-PC (■), Expanded NK-HD (□). Data shown here are representative of three independent experiments. **(E)** Xenograft mouse model was established by injecting MIA PaCa-2 tumor cells (1 × 10^7^ cells/mouse) subcutaneously into NSG mice. Ten days after cancer cell injection, *ex vivo* expanded NK cells (1 × 10^7^ cells/mouse) were injected intravenously every seven days. Tumor volume was measured by calipers every 3–4 days. Results shown are average tumor volumes offive mice per group ± SEM. Error bars represent ± SEM. Statistical differences between groups were calculated by Student's *t*-test (^***^p < 0.001).

We next determined if NK cell cytotoxicity against autologous and allogeneic PDAC tumor cells can be elevated by *ex vivo* activation and expansion processes. PBMCs were isolated from peripheral blood and co-cultured with IL-2 (500 U/ml) and gamma-irradiated feeder cell lines, according to the protocol published by Lim et al (Figure 4A) (29). A representative graph shows that enrichment of NK cell population, both in HD and patients with PDAC was comparable, up to 75% of total lymphocyte population after 10 days of *ex vivo* stimulation (Figure 4B). However, NK cells from patients demonstrated relatively smaller increase in cell numbers, compared to those from HD (292.2 ± 53.3 fold vs. 3010 ± 305.8 fold, respectively) (Figure 4C). The difference in fold expansion between HD and patients was statistically significant (Figure 4C), indicating that NK cells of patients with PDAC are less responsive to *ex vivo* stimulation. Regardless of poor expansion, activated NK cells of such patients demonstrated improved cytolytic function against autologous PDAC cells, showing up to 70% lysis at E:T ratio of 30:1 (Figure 4D). Furthermore, activated NK cells, regardless of autologous or allogeneic patients, showed significantly increased lysis in multiple PDAC cell lines including PANC-1, MIA PaCa-2, SNU-213, SNU-324, and SNU-410 (Figure S8). To further evaluate the efficacy of *ex vivo* activated NK cells in an *in vivo* model, we subcutaneously injected MIA PaCa-2 tumor cells (1 × 10^7^ cells/mouse) into immuno-compromised NSG mice. Ten days later when tumors began palpable, *ex vivo* activated NK cells (1 × 10^7^ cells/mouse) were intravenously injected every seven days. As seen in Figure 4E, tumor volume of NSG mice, adoptively transferred with *ex vivo* activated NK cells, was significantly smaller than that of control group over the 52-day observation period. These results indicate that *ex vivo* activated NK cells could be successfully trafficked to tumor sites and controlled the growth of PDAC tumors.

### NK Cell-Induced Cytotoxicity of PDAC Tumors Is Primarily Mediated by NKG2D and DNAM-1 Activating Receptors

**Figure 5:**
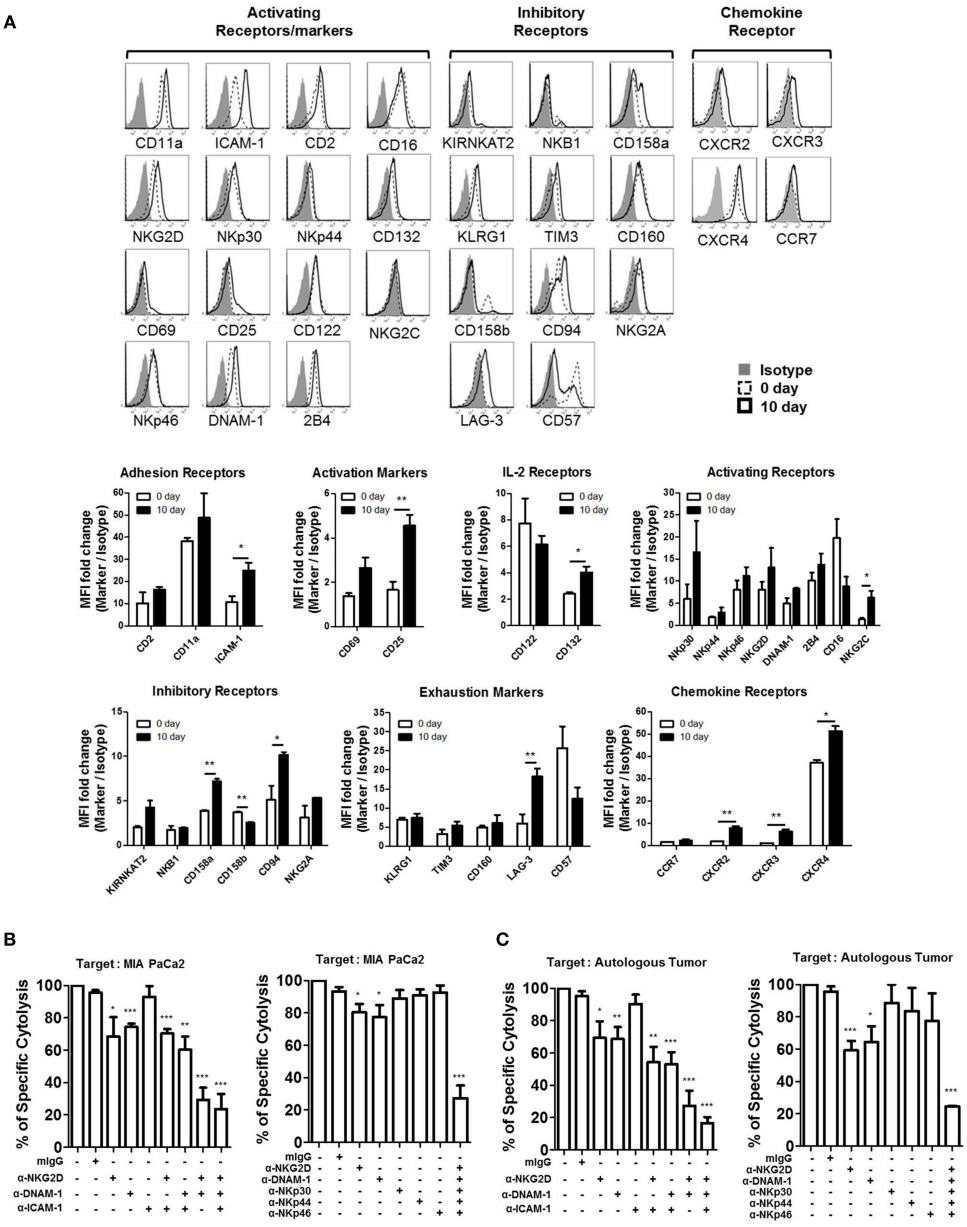
NKG2D and DNAM-1 are the main receptors responsible for killing autologous and allogeneic PDAC tumor cells. **(A)** Surface expression of multiple NK receptors was analyzed on CD3^−^CD56^+^ NK cells both prior to (dashed line), and 10 days after activation (solid line) using FACs. Isotype control is shown in gray. Data shown here are representative of five independent experiments. NK cell cytotoxicity assays against ^51^Cr-labeled MIA PaCa-2 **(B)** or autologous tumor cells **(C)**, pre-treated with 5 μg/ml of anti-NKG2D, anti-DNAM-1, and anti-ICAM-1, are shown. Specific lysis after 4 h was plotted at E:T ratio of 1:1. Surface expression of NKG2D and DNAM-1 ligands in each tumor targets was analyzed by FACS (Figure S7). Error bars represent ± SEM. Statistical significance was determined by one-way ANOVA (^*^p ≤ 0.05; ^**^p < 0.01; ^***^p < 0.001). Data shown here are representative of three independent experiments.

In order to identify the major receptors responsible for the lysis of PDAC cells, we screened the expression of surface receptors on patient-derived activated NK cells. Surface expression of adhesion receptors (CD11a, ICAM-1, CD2), IL-2 receptors (CD122, CD132), activating receptors (NKG2D, NKp30, NKp44, NKp46, DNAM-1, 2B4, CD16, CD69, NKG2C, CD25), inhibitory receptors (KIR NKAT2, NKB1, CD158a, CD158b, CD94, NKG2A), exhaustion markers (KLRG1, TIM3, CD160, LAG-3, CD57), and chemokine receptors (CCR7, CXCR2, CXCR3, CXCR4), before and after activation, is shown in Figure 5A. Among these, substantial up-regulation of ICAM-1 was noticeable while slight increase of its ligand LFA-1 is observed on activated NK cells. Since PDAC cells do not express LFA-1, LFA1/ICAM-1 interactions on NK-NK cells, not NK-PDAC cells, might have contributed to their intrinsic cytotoxicity leading to enhanced CTL functions (Figure 5A). Among chemokine receptors, up-regulation of CXCR2 and CXCR4 was evident, suggesting that activated NK cells could efficiently migrate toward PDAC tumors. In order to find the activating receptors responsible for lysing PDAC cells, blocking mAbs against ICAM-1, DNAM-1, NKG2D, and/or NCR (NKp46, NKp44, NKp30) were added to *in vitro* ^51^Cr-release assays using MIA PaCa-2 or autologous tumor cells as targets (Figures 5B,C). Although blockade of ICAM-1/LFA-1 interaction, using anti-ICAM-1 mAbs, did not alter the extent of tumor lysis, blocking NKG2D and DNAM-1 alone, or in combination, significantly lowered NK cytotoxicity up to 70% (Figure 5B, left). Interestingly, combination of all five antibodies (DNAM-1, NKG2D, NKp46, NKp44, and NKp30) reduced the killing of MIA PaCa-2 up to 85% (Figure 5B, right). Similar results were obtained with autologous tumor targets, highlighting the contribution of NKGD2 and DNAM1 in CTL lysis (Figure 5C). Taken together, these data indicate that NKG2D, DNAM-1, and NCR in combination, play major roles in NK cell-mediated killing of PDAC tumors.

### Fold Expansion of Peripheral NK Cells Upon *ex vivo* Activation Can Provide a Prognostic Factor for Overall Survival (OS) and Disease-Free Survival (DFS) of Patients With PDAC

**Figure 6:**
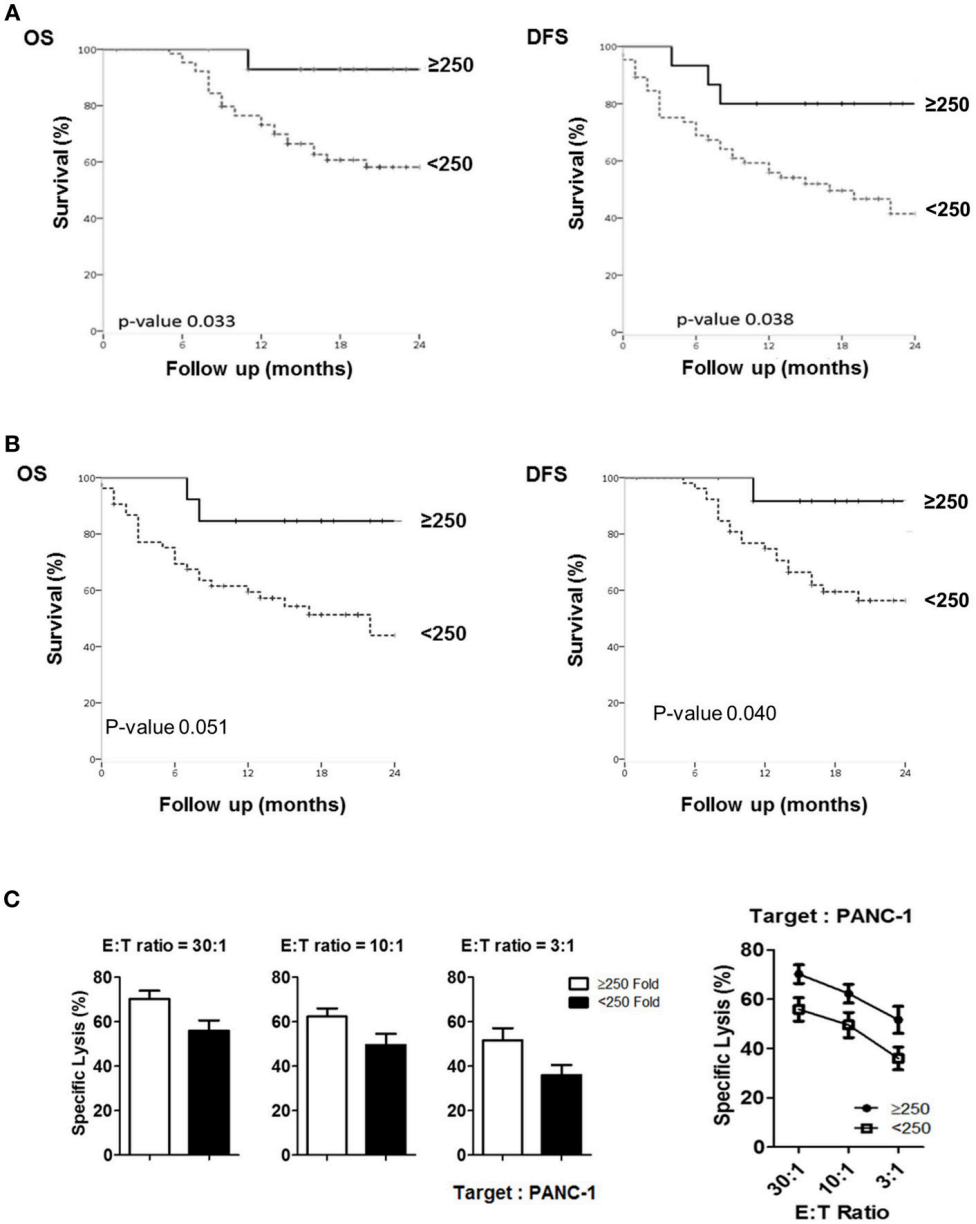
Comparison of actual 2-year survival rate and disease-free survival rate based on NK fold expansion rate. **(A)** The graphs of 2-year overall survival (OS) and disease-free survival (DFS) rates obtained from 80 patients are shown. (**B)** The graphs of 2-year overall survival (OS) and disease-free survival (DFS) rates obtained from 66 patients, excluding the patients treated with neo-CTX and/or neo-RTX are shown. **(C)** Tumor cytotoxicity of NK cells isolated from the patients who exhibited >250 and <250-folds.

To assess if NK cell expansion rate *ex-vivo* can provide a prognostic factor for PDAC patients, we first analyzed 5 different cutoff of expansion folds, 50, 100, 150, 200, 250 folds, obtained at day 10, day 14, and day 18 following NK cell activation in all patients (Figures S9–S11). Among these analyses, only 250 fold expansion rate analyzed at day 14 was found to be statistically different in their actual 2-year overall survival (OS) and disease-free survival (DFS) between HD and PDAC patients (Figure 6A). The median survival of the total enrolled patients was 16.5 months. The 2-year overall survival (OS) rate and median survival are significantly higher in the group showing NK expansion ≥250 fold than in those of <250 fold, with significant difference (92.9 vs. 58.2%; not reached vs. 27 months, respectively, *p* = 0.033). DFS rate and median DFS were also statistically significant (80.0 vs. 41.5%; not reached vs. 27 months, respectively, *p* = 0.038). Univariate analysis showed that higher *T* stage, positive nodal stage, status of resection margin, and higher NK fold change were associated with DFS and OS (Table 2). In a multivariate Cox proportional hazards model, higher T stage (hazard ratio [HR] 13458.169; 95% CI 0.000–1.611E + 193; *p* = 0.028) of T3 patients was a risk factor associated with poor survival in pancreatic cancer. Neither the presence of neoadjuvant therapies nor adjuvant therapies affected the OS nor DFS (Figure S12). Furthermore, these therapies did not affect the results obtained with 250 fold expansion rate (Figure 6B). Interestingly, patients' NK cells showing expansion rate ≥250 fold demonstrated higher tumor cytotoxicity than those of <250 fold (Figure 6C). Taken together, these data suggest that *ex vivo* expansion of NK cells could provide a therapeutic modality, whereas their fold expansion could be a potentially significant prognostic indicator of OS and DFS in such patients.

**Table 2:** Univariate and multivariate analyses of prognostic factors.

**Prognostic factors**						
		**Univariate analysis**		**Multivariate analysis**		
**Factors**		***n*** **(median survival)**	***p-*****value**	**HR**	**95%CI**	***p*****-value**
Age		67	0.810			
Sex	M	49 (29)	0.097	1.734	0.727–4.135	0.214
	F	31 (NR)				
T stage	1	3	<0.001			0.028
	2	5				
	3	71		13458.169	0.000–1.611E + 193	
	4	1				
N	stage 0	29 (NR)	0.054	1.971	0.734–5.292	0.178
	1	51 (29)				
M	stage 0	78 (29)	0.559			
	1	2 (8)				
R	status 0	68 (29)	0.014	1.875	0.806–4.364	0.145
	1	12 (12)				
NK fold change	<250 ≥250	65 (27) 15 (NR	0.033	0.329	0.043–2.536	0.286
neo CTx. (N/Y)		66/14 (NR/27)	0.816			
neo RTx. (N/Y)		73/7 (29/27)	0.199			
adj CTx. (N/Y)		22/58 (NR/29)	0.919			
adj RTx. (N/Y)		66/14 (29/27)	0.455			

*NR, not reached; neo, neoadjuvant; adj, adjuvant; CTx, chemotherapy; RTx, radiotherapy*.

## Discussion

TIL provide important information regarding the prognosis and survival of patients with pancreatic tumor. Infiltration of CD4^+^ and CD8^+^ T cells correlated with a higher rate of disease-free survival and improved prognosis in the patients with PDAC (15, 38, 39) while increased leukocyte infiltration into myeloid-derived suppressor cells (MDSC), TAM, and Treg paralleled disease progression. In this study, we found that unlike CD8^+^ T and CD4^+^ T cells, pancreatic tumor cells are selectively resistant to NK cell-mediated immune surveillance. The very low frequency (<0.5%) of NK cells in tumors is a unique feature of patients with pancreatic cancer, as TILs isolated from patients with other solid tumor malignancies (breast, liver, lung, stomach, and colorectal cancers) show 3–10% of NK cell infiltration (40–45). Further in-depth analysis revealed that NK cells in patients with PDAC express significantly reduced CXCR2 on their surface, rendering them incapable of trafficking toward PDAC. In addition, poor engagement of NKG2D and DNAM-1 activating receptors in patients' NK cells explain the impaired killing of PDAC tumor cells. More importantly, circulating NK cells, upon reaching the hypoxic tumor microenvironment (pO_2_ ≤ 1.5%) failed to survive or proliferate, thereby contributing to overall specific immune escape of NK cells in such patients.

Ours is the first report to demonstrate multiple NK cell-associated defects in newly developed patients. One of the major findings here is that peripheral NK cells lose the surface expression of CXCR2, which binds to CXCL chemokines released from PDAC cells. Circulating NK cells, however, do express CXCR4, but its ligand CXCL12 was not secreted by PDAC tumors. Hence, there are no apparent chemokine signals that could attract circulating NK cells to the PDAC tumors. Of note, Kremer et al. (46) reported that NK cells lose surface CXCR2 upon *ex-vivo* activation and tumor localization. In contrast, we found low CXCR2 in NK cells from the periphery and impaired NK cell localization within PDAC tumors, despite high abundance of its ligand CXCLs within tumors. The discrepancy between the two reports could have been attributed to a number of factors. Compared to RCC, PDAC tumor cells might release higher level of immunosuppressive cytokines, TGF-β or IL-10 (47), and downregulate CXCR2 on NK cells in the peripheral blood. In addition, Kremer V et al. used IL-2 plus LCL while we used IL-2 plus LCL and KL1 two feeder cell lines. Therefore, strong cytotoxic signals generated by two feeder cells in our protocol might override the IL-2-initiated CD182 downregulation seen on NK cells of RCC patients.

Unlike CXCR2, poor recognition of PDAC cells by NK cells was not due to systemic immune suppression in the patients, since those isolated from HD also failed to kill the tumor cells. However, activated NK cells from both patients and HDs could efficiently kill PDAC cells, in both autologous and allogeneic settings. This enhanced killing was mediated by cooperation of multiple NK-activating receptors, primarily NKG2D and DNAM-1, as well as NKp30, NKp44, and NKp46. These data suggest a possible underlying mechanism for the low frequency of NK cells within PDAC tumors. Therefore, defective localization, together with impaired tumor cytotoxicity, contributes to the lack of NK cell function within the PDAC tumors. Unfortunately, the number of NK cells within TILs was too low for further phenotypic analysis. However, among the subpopulation of CD3^−^/CD8^−^ TILs that could be analyzed, we found poorly cytotoxic CD27^+^/CD56^dim/bright^ NK phenotype (Kwon et al., unpublished data). Typically, CD27 and CD28 are required for the generation and long-term maintenance of T cell immunity. However, previous reports demonstrated that CD27^+^ NK cells were mostly CD56^dim/bright^, had lower levels of perforin and granzyme B, with low cytolytic potential (48). These findings imply the presence of additional NK functional defects within the tumor microenvironment of patients. Increased expression of MMP-9 and IDO by pancreatic tumor cells has also been shown to impair NK cytotoxicity (49). Therefore, NK cells within PDAC are probably subject to multiple layers of immunosuppressive mechanisms.

Lastly, NK cells, if at all localized within PDAC tumors, would have encountered gradually decreasing O_2_ concentrations within the tumor microenvironment. Although hypoxia is a common feature of several solid tumors, pancreatic cancer is found to be the most hypoxic (50), the major mechanism being associated with insufficient and aberrant blood vessel supply. Hypoxia is also a strong activator of pancreatic stellate cells, the major fibroblastic cells of the pancreas, thereby facilitating their fibrosis, which limits the access of conventional chemo-drugs (51). We found that resting NK cells, exposed to hypoxic condition of <1.5% pO_2_, failed to expand, while those in 20% pO_2_ expanded exponentially within 18 days following initial culture. Therefore, pre-activation of NK cells is strongly recommended for enabling tumor recognition and efficient cytolysis within the hypoxic microenvironment of pancreatic tumor.

In a recent phase III study (CONKO-001), adjuvant Gemcitabine, after surgical resection, could achieve prolonged median survival of 22.1 vs. 20.2 months, and over 20% 5-year survival rate in patients with PDAC (52). Although the precise mechanism underlying the efficacy of Gemcitabine is not completely understood in this trial, anti-tumor responses, observed by Gemcitabine, was found to be primarily associated with activation and localization of NK cells in PDAC tumors (53). When newly established transgenic mice with resectable PDAC, LSL-KrasG12D × p53^fl/fl^, was co-delivered with a transposon encoding constitutively active Akt2, they developed a single pancreatic nodule with histopathologic features of human PDAC (53). Gemcitabine administration after resection surgery significantly decreased infiltration of CD11b^+^Gr1^int^F4/80^int^ MDSCs at the boundary of the resection site while increasing infiltration of NK cells. Depletion of NK cells, but not T cells, abrogated Gemcitabine-induced anti-tumor effects, suggesting NK cells as important effectors following adjuvant-therapy in pancreatic cancer. Similarly, a patient exhibited regression of several pancreatic cancer metastases following administration of Ipilimumab (anti-CTLA4 mAbs). Upon analysis of the patients' TILs, it was found that CD56^bright^CD16^−^ NK cells were the major components responsible for killing pancreatic tumor targets, as well as for secretion of IFN-γ and TNF-α (54). Although the precise mechanism underlying NK cell infiltration and activation remained unclear in these studies, it was speculated that activation of CD4^+^ T cells by anti-CTLA4 mAbs caused release of surplus IL-2 and stimulation of circulating NK cells, which could have trafficked to PDAC tumor microenvironment. Our data support this hypothesis, since resting NK cells up-regulated CXCR2 upon IL-2 stimulation, and promoted migration toward PDAC tumors. Therefore, NK cell activation and tumor trafficking appear to be critical for the prognosis of patients with pancreatic tumor. In line with these findings, we discovered that patients manifesting over 250-fold NK cell expansion *ex vivo* demonstrate prolonged overall survival and significantly higher disease-free survival. These results are of particular importance, since this method provides a non-invasive straightforward tool to determine OS and DFS while providing a therapeutic option amenable to *in vivo* NK cell adoptive transfer for patients with PDAC. Therefore, our data propose possible underlying mechanisms for multiple NK defects in patients with PDAC, and further provide a therapeutic modality and useful diagnostic tool for assessing disease-free survival rate in such patient.

## Ethics Statement

The use of Healthy donor blood: 1040548-KU-IRB-16-103-A-2 and of cancer patient sample: SNUH 1503-093-657.

## Author Contributions

S-JK, J-YJ, and K-ML designed the experiments. SL, JK, and SJ performed most of the experiments, analyzed data, prepared figures, and collaborated in manuscript writing. MS, JK, T-JK, KI, YH, WK, S-WK, and CY contributed to study design, analysis of results, and corrected the manuscript. S-JK, J-YJ, and K-ML conceived and supervised the study and wrote the manuscript.

### Conflict of Interest Statement

The authors declare that the research was conducted in the absence of any commercial or financial relationships that could be construed as a potential conflict of interest.
