# Supplementary material for: Defective Localization With Impaired Tumor Cytotoxicity Contributes to the Immune Escape of NK Cells in Pancreatic Cancer Patients
Source: Front Immunol. 2019 Apr 9;10:496. doi: 10.3389/fimmu.2019.00496 (PMC6465515; doi:10.3389/fimmu.2019.00496)
Supplement: Supplementary file 1 [file Data_Sheet_1.docx]

*Supplementary Material*

**Defective localization with impaired tumor cytotoxicity contributes to the immune escape of NK cells in pancreatic cancer patients**

Seon Ah Lim^1^, Jungwon Kim^1^, Seunghyun Jeon^1^, Min Hwa Shin^1^, Joonha Kwon^1^, Tae-Jin Kim^1^, Kyungtaek Im^1^, Youngmin Han^2^, Wooil Kwon^2^, Sun-Whe Kim^2^, Cassian Yee^4^, Seong-Jin Kim^3,*^, Jin-Young Jang^2,*^ and Kyung-Mi Lee^1,4,5,*^

*** Correspondence:** Kyung-Mi Lee : kyunglee@korea.ac.kr

Jin-Young Jang : jangjy4@gmail.com

Seong-Jin Kim : jasonsjkim@snu.ac.kr

**Supplementary Table S1**

| **Gene** | **Forward primer (5’-3’)** | **Reverse primer (5’-3’)** |
| --- | --- | --- |
| **CXCL1** | GAA AGC TTG CCT CAA TCC TG | CTT CCT CCT CCC TTC TGG TC |
| **CXCL2** | GGG CAG AAA GCT TGT CTC AA | GCT TCC TCC TTC CTT CTG GT |
| **CXCL3** | CGC CCA ACC CGA AGT CAT AG | GCT CCC CTT GTT CAG TAT CTT TT |
| **CXCL5** | GGA AGG ATT TTG TTG TTG TT | AGT CAC CTA CAA TTC AAG AC |
| **CXCL6** | AGA GCT GCG TTG CAC TTG TT | GCA GTT TAC CAA TCG TTT TGG GG |
| **CXCL7** | TGG AAA CAA CTC TAG CTC AGC CTT CTC | TCC AGG CAG ATT TTC CTC CCA TCC |
| **CXCL8** | ACA CTC CAC ACC TTT CCA T | GGC ACA CCT CAT TTC CAT TG |
| **CXCL12​** | ATG CCC ATG CCG ATT CTT CG | GCC GGG CTA CAA TCT GAA GG |

**Table S1.** List of Primers used for Quantitative Reverse Transcription polymerase Chain Reaction

**Supplementary Figure S1**

**
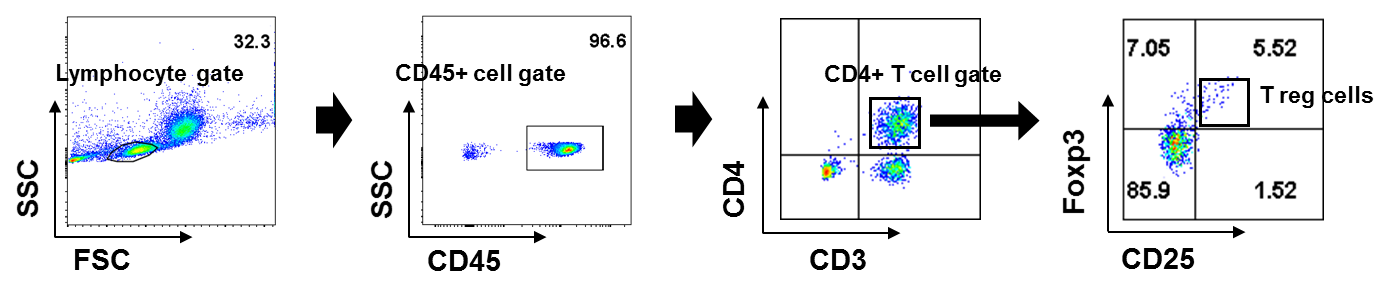
**

**Figure S1.** Gating strategies shown for the detection of CD4+ T cells and Tregs using FACS

**Supplementary Figure S2**


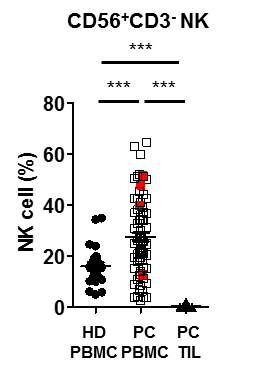


**Figure S2.** The percentages of lymphocytes from individual patients are summarized as dot plots The peripheral NK cell percentages of patients with respect to their NK cells infiltrated within TIL was marked in Red. Statistical differences between each group were calculated by Student’s *t-* test (*, p ≤ 0.05; **, p < 0.01; ***, p < 0.001).

**Supplementary Figure S3**


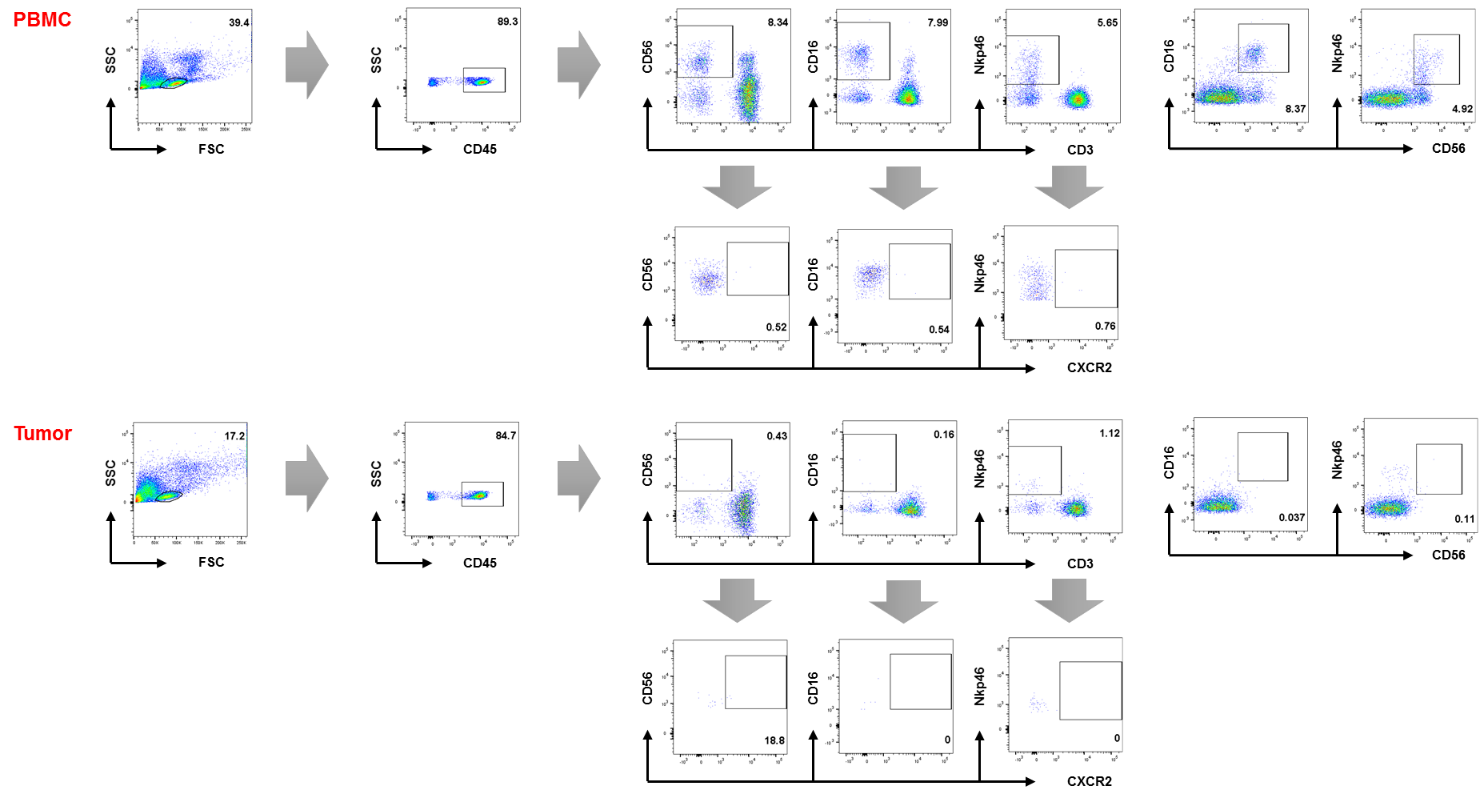


**Figure S3.** Gating strategies shown for the detection of CD3-CD56+, of CD3-CD16, of CD3-NKP46+, and CD56+CD16+ NK cells using FACS

**Supplementary Figure S4**

**
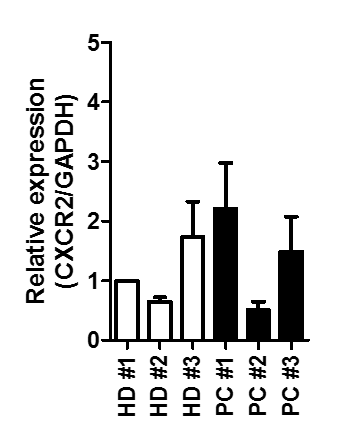
**

**Figure S4.** mRNA levels of CXCR2 in NK cells from HD and PC PBMC was measured by qRT-PCR

**Supplementary Figure S5**


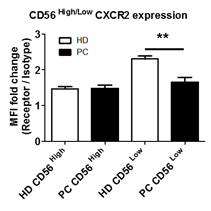


**Figure S5.** MFI of CXCR2 was calculated and plotted as bar graph. CD56+NK cells were divided by CD56high and CD56low NK cells populations. (CXCR2 MFI/Isotype control MFI)

**Supplementary Figure S6**

**
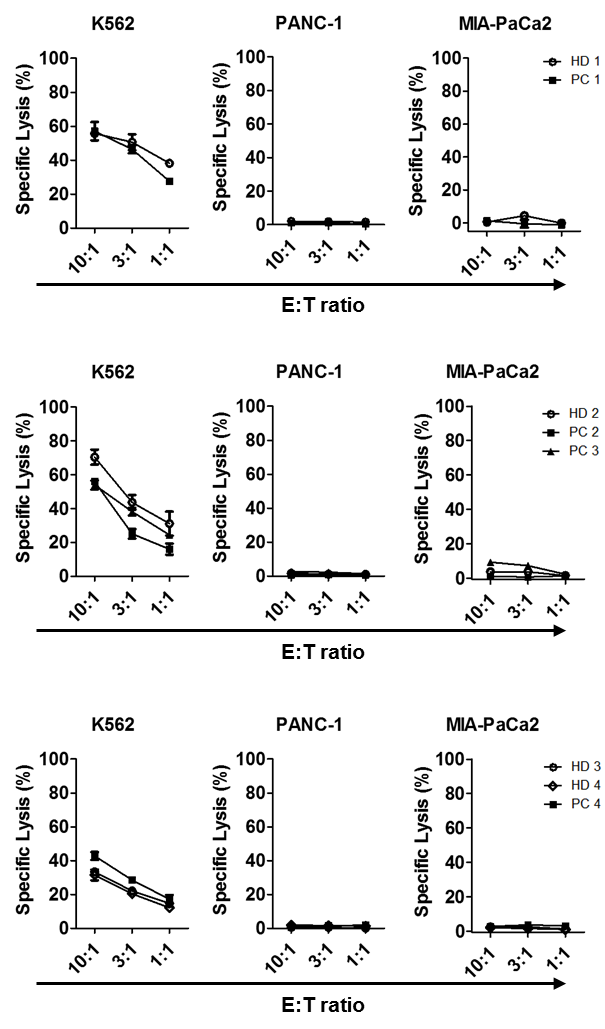
**

**Figure S6.** ^51^Cr-release assay results against K562, PANC-1, and MIA-PaCa2 using highly purified resting NK cells from HD and Patients

**Supplementary Figure S7**


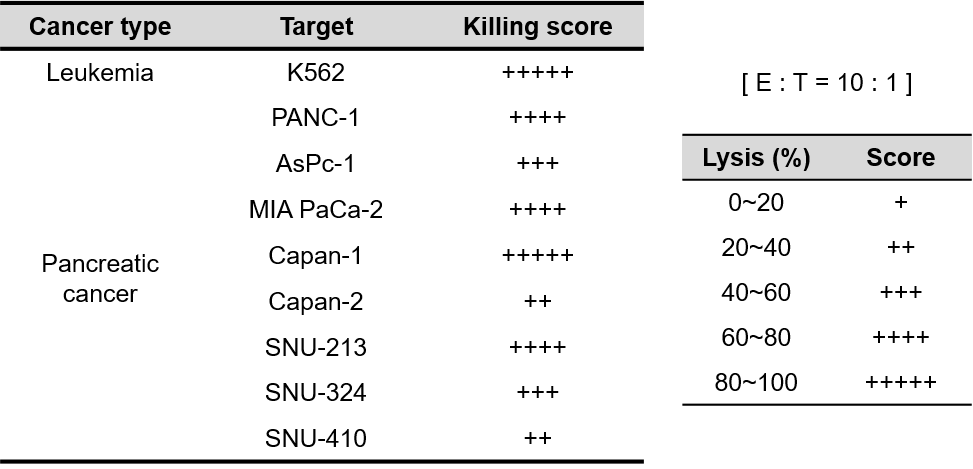


**Figure S7.** Evaluation of the cytotoxicity of allogeneic NK cells against pancreatic cancer cell lines using Chromium-51(^51^Cr) release assay(CRA)

**Supplementary Figure S8**

**
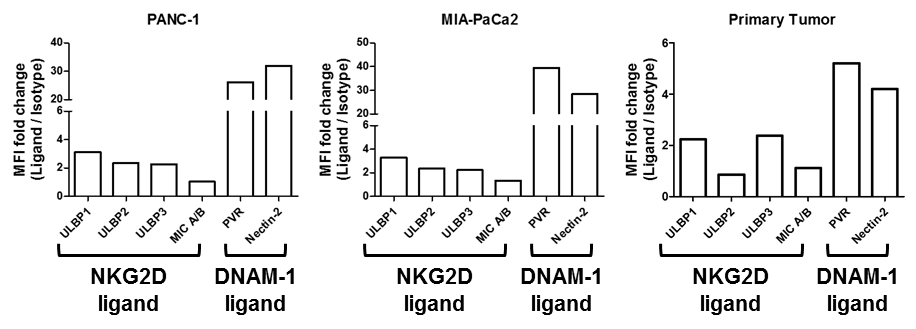
**

**Figure S 8.** Ligand expression of NKG2D and DNAM-1 of PDAC cell lines was analyzed by FACS

**Supplementary Figure S9**

**
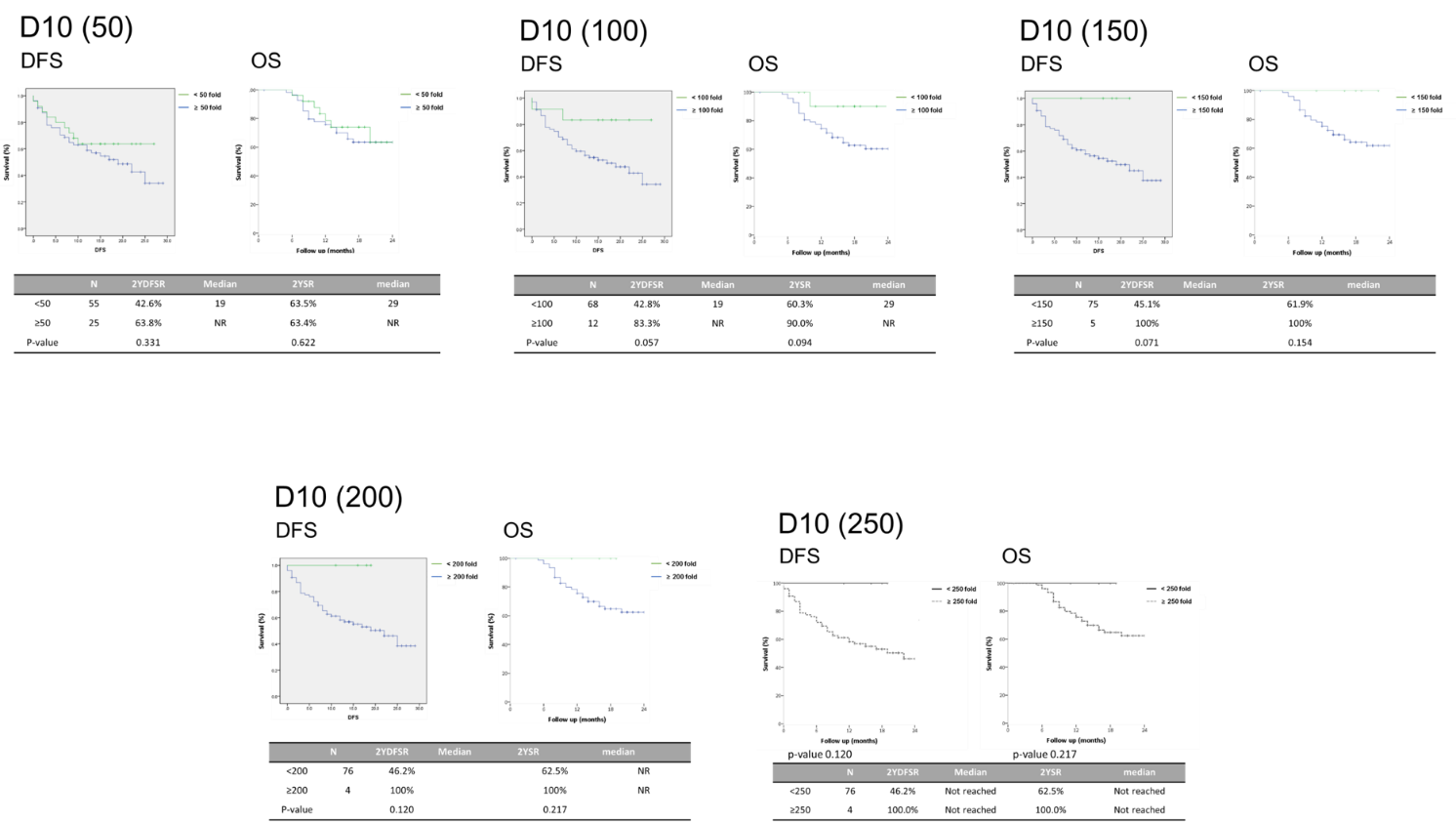
**

**Figure S9.** Survival rates of patients whose NK cells proliferated 50, 100, 150, 200 and 250 fold at Day10 of culture are shown

**Supplementary Figure S10**

**
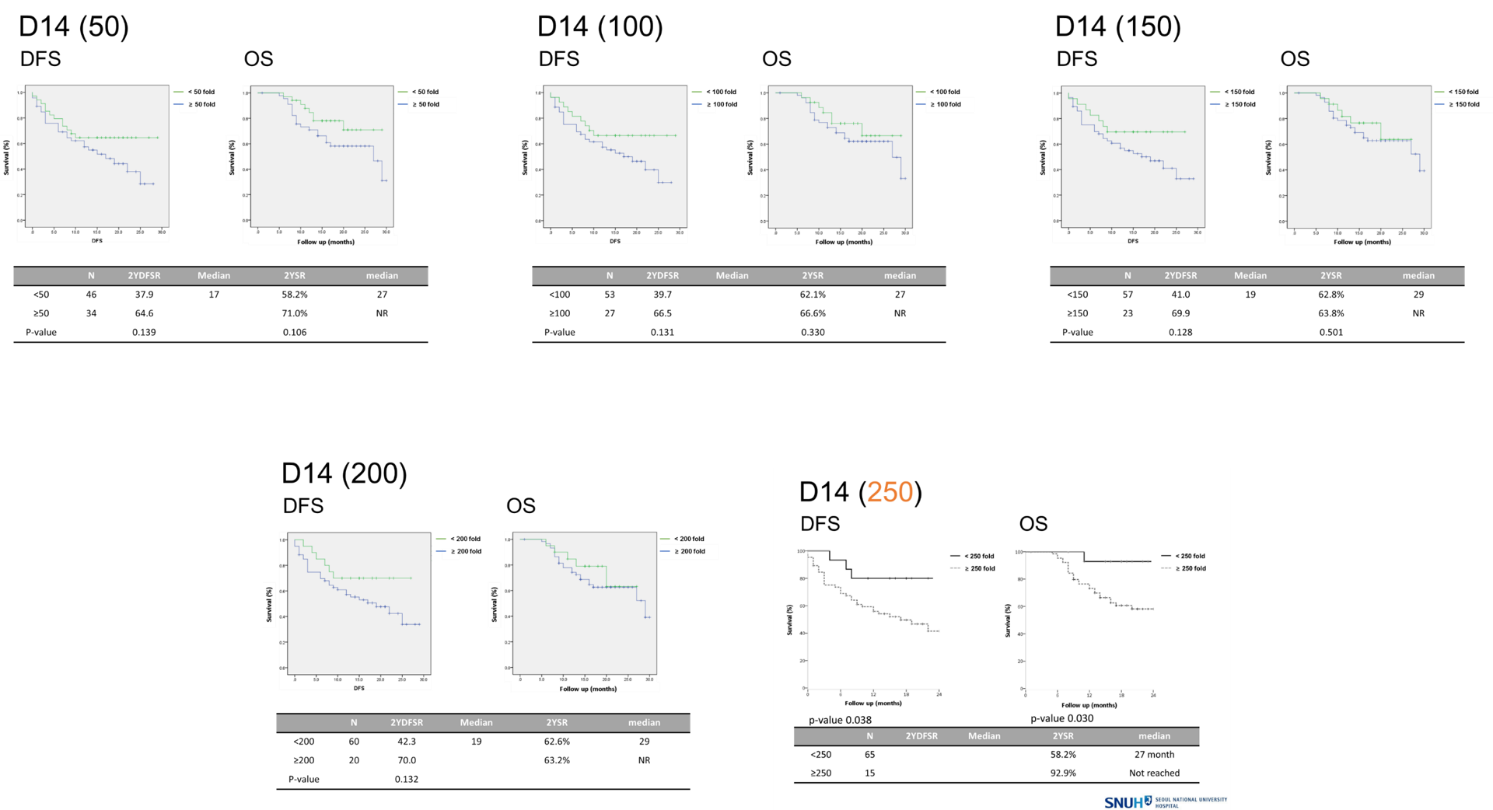
**

**Figure S10.** Survival rates of patients whose NK cells proliferated 50, 100, 150, 200 and 250 fold at Day14 of culture are shown

**Supplementary Figure S11**

**
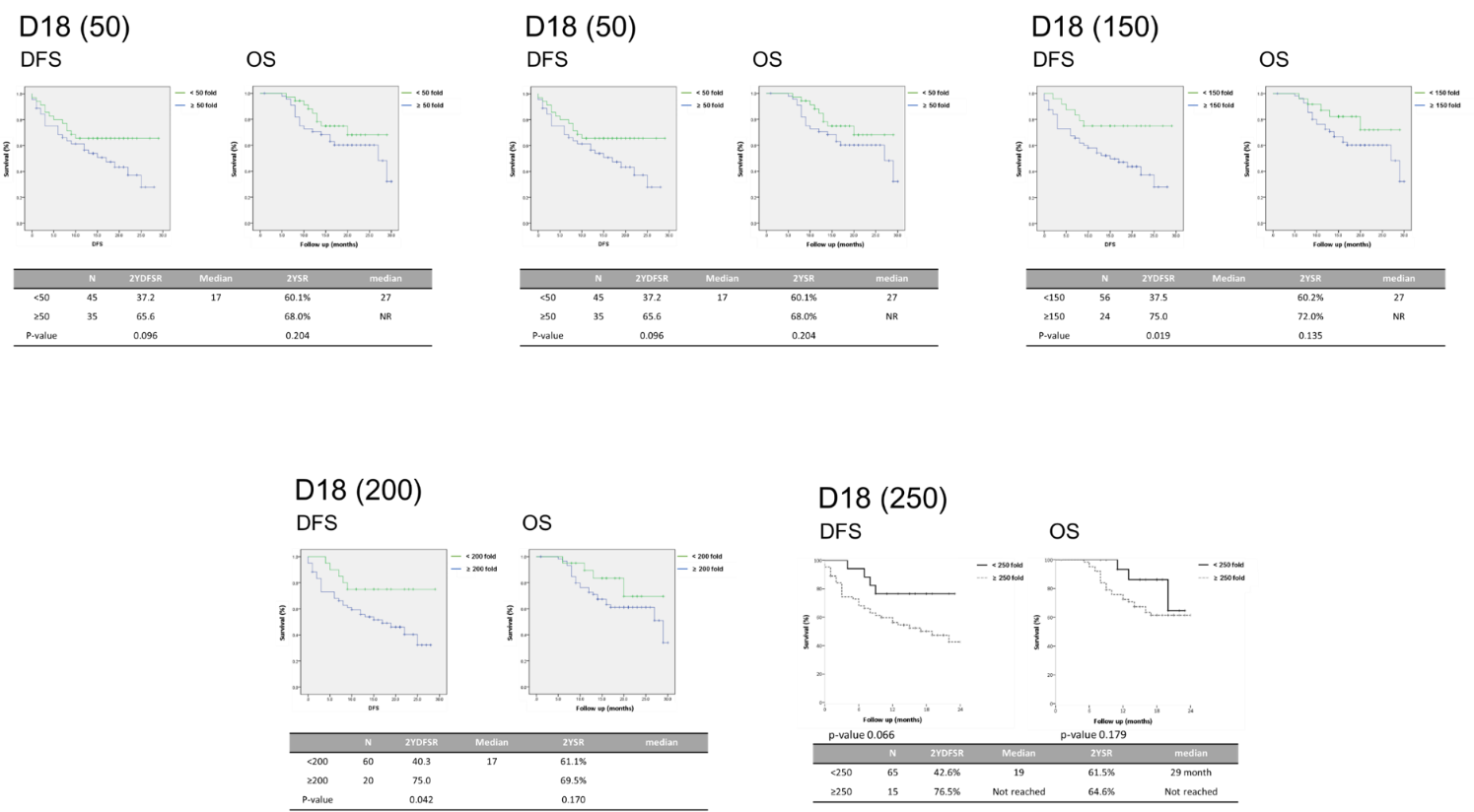
**

**Figure S11.** Survival rates of patients whose NK cells proliferated 50, 100, 150, 200 and 250 fold at Day18 of culture are showed

**Supplementary Figure S12**

**
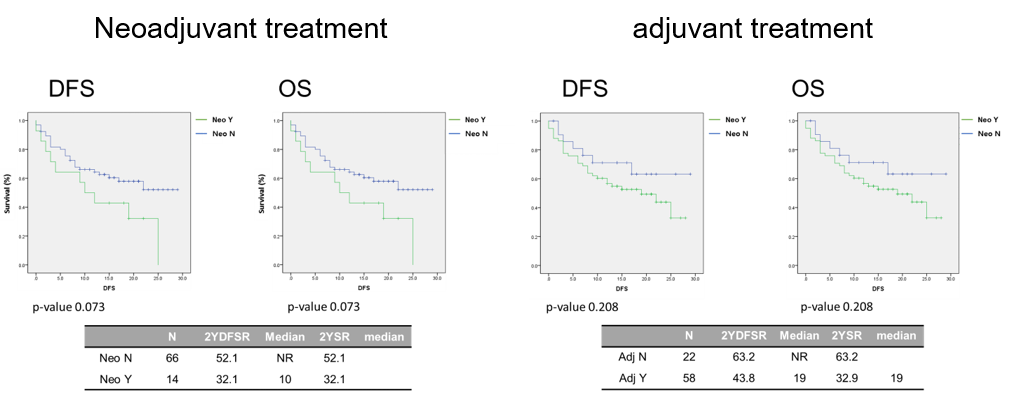
**

**Figure S12.** Survival rates of patients with neoadjuvant or adjuvant chemotherapy are shown.
